# Translating training to medical practice in trauma care, a literature review

**DOI:** 10.1007/s00068-024-02548-1

**Published:** 2024-05-31

**Authors:** Alexandra Haută, Radu-Alexandru Iacobescu, Mihaela Corlade-Andrei, Paul Lucian Nedelea, Carmen Diana Cimpoeșu

**Affiliations:** 1 Department of Surgery II-Emergency Medicine, University of Medicine and Pharmacy “Grigore T. Popa”, Iași, Romania; 2 Department of Medicine II-Nursing, University of Medicine and Pharmacy “Grigore T. Popa”, Iași, Romania; 3 Department of Thoracic Surgery, Pulmonary Disease University Hospital, Iași, Romania; 4 Department of Emergency Care, “Sf. Spiridon” University Emergency Hospital, Iași, Romania

**Keywords:** Trauma training, Trauma management, Adult, Emergency medicine, Education, Program evaluation, Simulation, Wounds and injuries

## Abstract

Trauma, a global health challenge, remains a significant cause of mortality despite advances in trauma management. The establishment of trauma teams has revolutionized care in trauma resuscitation. The training of these teams is designed to promote self-trust and empower trainees in trauma care, enhance performance, and improve patient outcomes. Various training curricula have been developed, utilizing a plethora of teaching methods such as lectures, simulations, debriefings, skill workshops, and demonstrations. However, a universally accepted gold standard curriculum in trauma training is yet to be defined, and there is no standard method for delivering education in injury care teaching. In this review, we have examined relevant literature data on standard teaching programs, the educational delivery methods used, and their impact on adult trauma patients’ outcomes and trained team-related outcomes. While most studies indicate improved trained team performance, they consistently show no improvement in patient-specific outcomes such as mortality, morbidity, and length of stay. However, data hints at optimal educational delivery and the role that technology may play in the future of trauma training development.

## Introduction

Trauma is a global concern due to its high fatality rate and disability in all geographic regions. World Health Organisation (WHO) reports that 8% of deaths were caused by trauma (over 4 million people) in 2019 and that trauma is the first cause of death for people under the age of 45 in developed countries [1]. Implementation of effective trauma systems adapted to available resources is detrimental to the augmentation of resuscitation outcomes, as estimates show that up to two million lives are needlessly lost each year due to inadequate trauma systems and resource constraints [2]. The trauma system was pioneered in the 1970s by the American College of Surgeons. This initiative revolutionized trauma care by organizing trauma centers based on available infrastructure, material capabilities, and human resources, which resulted in the classification of four levels of trauma care [3]. The implementation of this system and further optimization efforts have had a substantial impact on patient mortality and morbidity rates [4]. Following their lead, other countries have adopted similar systems with data showing reproducible results in various settings [5, 6].

Providing care for the injured is a multifaceted process that involves several interconnected phases: the prehospital stage, transportation logistics, and ultimately, intrahospital care facilitated by trauma team activation [3]. Thus, care outcomes are highly dependent on all participants that compose each stage of care. Accurate field triage and prompt trauma activation upon arrival at the trauma scene by the intervention team and prompt management of life-threatening hemorrhagic injuries play a pivotal role in prehospital care and determine the outcome of the resuscitation [7, 8]. Further care is then provided in hospitals by well-orchestrated specialized trauma teams. Trauma teams have been the heart of trauma system development. They consist of a multidisciplinary team comprised of medical specialists, nurses, and paramedics involved in well-coordinated simultaneous procedures [9]. Evidence has shown that trauma team implementation improved patient outcomes [10]. However, there is debate about the impact further training of trauma teams has on patient outcomes [11].

Trauma resuscitation is time-sensitive, and the ”golden hour” for injury care is crucial for resuscitation results [12]. Outcome often depends on the emergency staff’s level of education and cohesion in a critical situation [13]. Despite the experience level, resuscitation is seldom without error [14]. Team strength does not rely solely on individual skills in injury management, commonly known as technical skills, but rather on the compliance of individuals to collaborate and communicate in an emergent situation and the ability to self-reflect on each experience, coined non-technical skills [15]. Trauma team training (TTT) faces numerous challenges to encompass both skill sets and coherently cover all staff participants present in trauma resuscitation [16]. Considering trauma team establishment’s significant impact on injury care, trauma team training has received administrative support [9, 17]. Yet, there is current lagging in global implementation as an international survey has shown that 39% of hospitals do not have a trauma team in their hospital, and in the ones that had a team organized only 69% had a dedicated trauma team, and further, formal training was provided just by 33% of the hospitals [18].

To date, many formal educational programs have been implemented for TTT. However, little is known about the impact these have on patient outcomes. In this narrative review, we evaluate common teaching methods, TTT programs, and literature data about how they impact patient outcomes.

## Methods

Between October and December 2023, we performed a PubMed database survey using key terms such as Trauma Team, Training, Education, Simulation, Patient Outcomes, and Patient Care. Relevant articles were screened to report patient outcomes, training outcomes, or both as applied to in-hospital trauma management. We did not include studies related to military trauma care or studies involving prehospital care. Data about the training program used, teaching methods, training participants, and outcomes considered were extracted, commented on in the text below, and schematically presented in Table 1. We discussed these data, grouping them into relevant categories such as training methods and curricula, trained team-related outcome measures, and patient-related outcome measures.

**Table 1:** Trauma team training and correlation with training outcomes

First Author [ref.]; Study Design; Setting (year).	Study sample	Intervention, timing, and teaching methods	Outcome measures studied	Findings
Kristiansen [22]; Prospective, Interventional, Uncontrolled, Unblinded, Pre/Post-Test; Level 2 Regional Hospital (2020).	Between 84 and 94 health professionals	Six sessions of ATLS every two months. One 90-minute lecture, two off-site simulation scenarios with debriefings.	Time to chest radiography Time to CT Time to CT interpretation Time to ward Time to OR	No significant reduction in total processing time.
Park [47]; Retrospective, Pre/Post-Test; Level 1 Trauma Center (2020).	Residents	Technical skills and ATLS trauma management algorithm. Procedural training and simulations	Time to intervention: intubation, tube thoracostomy, vascular access, interosseous access, arterial line, REBOA device, pelvic binder, resuscitative thoracotomy, CT, OR.	No improvement in time to CT (32.5 to 30.5 min, *P* = 0.36). Significant improvement in time to thoracotomy (14 to 3 min, *P* = 0.02), thoracostomy (13 to 6 min, *P* = 0.04), and percutaneous sheath access.
Long [12]; Prospective, Interventional, Uncontrolled, Unblinded, Pre/Post-Test; Level 1 Trauma Center (2019).	Residents ED nurses Respiratory therapists Radiology technicians Paramedics	ATLS trauma management algorithm. Five in-situ simulation training scenarios with debriefings.	Time to primary survey Time to secondary survey Time to CT Time to OR	No significant reduction in time to primary survey. Time to secondary survey decreased (14 to 6 min, *P* = 0.004). Time to CT decreased (23 to 16 min, *P* = 0.01).
Hong [25]; Retrospective, Pre/Post-Test; University-affiliated Hospital (2018).	ED Doctors ED Residents ED nurses	Proprietary resuscitation algorithm. In-situ simulations, demonstrations, video-demonstrations.	Time from arrival to test/procedure: green channel open, cervical collar, venous line establishment, first fluid administration, oxygen delivery, artificial airway establishment, central venous catheter, chest tube insertion, chest band, urinary catheter, hemostasis, blood routine report, other blood tests, CT scan, X-ray, ultrasound, electrocardiogram, consultation call, trauma team arrival, packed red blood cell preparation and transfusion, hemostatic administration, analgesics, ED departure.	Significant post-test reduction of time until routine blood test (13 to 8 min, *P* < 0.01), initiation of hemostasis (113.5 to 31 min, *P* = 0.01), time to CT (58.5 to 29.5 min, *P* = 0.01) and time to Tranexamic Acid administration (90 to 31 min, *P* < 0.01).
Knobel [26]; Retrospective, Pre/Post-Test; Level 1 Trauma Center (2018).	190 participants: 17 traumatology doctors, 60 anesthesiology doctors, 40 Emergency and anesthesia nurses, 10 radiology doctors.	24 sessions of ALTS, monthly. In-situ simulation and video-assisted debriefings.	Time to CT/OR Participants confidence level	Significant reduction in time to CT (22.3 to 18.6 min, *P* = 0.001). Total resuscitation time decreased. Self-confidence and knowledge increased.
Murphy [21]; Retrospective, Pre/Post-Test; Level 1 Trauma Center (2018).	324 multidisciplinary trauma team staff.	Proprietary trauma resuscitation algorithm. Three 60-minute lectures and four simulation scenarios followed by debriefings.	Time to OR Mortality Time to discharge from ED (LOS)	Significant reduction in time to OR (2.63 to 0.55 h, *P* < 0.001). No significant improvement in mortality rates. LOS increased post-intervention (4.88 to 7.17 h, *P* < 0.001) except for patients requiring surgery.
Malekpour [40]; Retrospective, Pre/Post-Test; Three Non-Trauma Rural Hospitals (2017).	Not available	Eight-hour RTTDC training. The teaching method is not specified.	Time to transfer acceptance Transfer time LOS Complications Mortality	Time to transfer acceptance and total transfer time decreased (139.2 to110 min, *P* = 0.003 and 257.3 to 219.2 min, *P* = 0.002, respectively). No improvement in LOS, complications, or mortality.
Petroze [48]; Retrospective, Pre/Post-Test; University Hospital (2015).	24 Faculty surgeons, 15 Trauma Nurses, 25 Faculty residents and nurses.	Three-day of ATLS, and Canadian Network for International Trauma Team Training Course. Demonstration courses.	Mortality	Mortality was reduced from 8.8–6.9%, but it was no statistically significant *P* = 0.11. Mortality was significantly reduced in the subgroup with reduced GCS 3–8 subset (58.51–37.10%, *P* = 0.009).
Steinemann [23]; Prospective, Interventional, Uncontrolled, Unblinded, Pre/Post-Test; Level II Trauma Center University Teaching Hospital (2011).	123 participants Multidisciplinary physicians Residents Nurses Respiratory Therapists ED technicians	Crisis team training course. One-hour online presentation, three-hour high-fidelity in-situ simulation, and video-assisted debriefings.	Teamwork assessment T-NOTECH score Task completion number Time to primary survey	Improved teamwork scores (T-NOTECH scores 16.7 to 17.7, *P* < 0.05). 18% reduction in ED resuscitation time (32 to 26 min, *P* < 0.05). The task completion rate increased (48 to 62 participants, *P* < 0.001). No improvement in mortality, morbidity, or LOS. Improved time to primary survey.
Kappel [44]; Longitudinal Cohort Study; Level III and Level IV Trauma Centers (2011).	Medical personnel	RTTDC and Comunication module. On-site training and communication instruction sessions.	Time to decision to transfer Time to finding an accepting facility Time to transfer squad arrival	Time to decision to transfer significantly improved in the trained cohort (114.35 vs. 95.72 min exposed to RTTDC and 77.17 min exposed to RTTDC and communication module, respectively, *P* < 0.05). The time to transfer squad arrival was shorter in the cohort that received communication training (31.12 vs. 67.19 min for RTTDC cohort, *P* < 0.01).
Capella [24]; Prospective, Interventional, Uncontrolled, Unblinded, Pre/Post-Test; Level 1 Trauma Center (2010).	Surgeons Nurses Residents	Seven sessions of proprietary trauma training algorithm and TeamSTEPPS. Two-hour didactic sessions, two-hour simulation sessions, and videotape feedback.	TPOT score Mortality Complications Hospital LOS ICU LOS Time to FAST Time to CT Time to OR Time to intubation ED LOS	Team performance scores improved (3.12 to 3.70, *P* < 0.001). Significant improvement of time to CT (26.4 to 22.1 min, *P* = 0.005), intubation (10.1 to 6.6 min, *P* = 0.049), and OR (130.1 to 94.5 min, *P* = 0.021). No improvement in mortality, complication rate, LOS measurements, and time to FAST.

ATLS- Advanced Trauma Life Support, CT- Computed Tomography, ED- Emergency Department, LOS- Length Of Stay, ICU-Intensive Care Unit, FAST-Focused Assessment Sonography in Trauma, REBOA- Resuscitative Endovascular Balloon Occlusion of the Aorta, RTTDC- Rural Trauma Team Development Course, TPOT-Trauma Team Performance Observation Tool

## Training methods and curricula

A standard for trauma team training has not yet been defined. There is variability between care providers regarding curriculum contents and training methods employed (Table 1). Conventional education in trauma consisted previously of competency acquisition with experience gained from working in a trauma department without formal training [13]. The robustness of the developing trauma system needed to convey to trauma resuscitation participants new practical skills and the capacity to function effectively within a team, thus, new teaching methods were required. TTT usually employs a hybrid educational approach utilizing various methods, from classroom teaching to immersive simulation scenarios followed by structured debriefings [19].

Lecture-based teaching has been the norm in trauma education and has some benefits, like accessibility for a larger number of participants and low cost [15, 20]. Still, it is limited by the lack of participant engagement and the limited retention of information [15]. Several studies report the use of lecture-based teaching, with session durations varying from 60 to 120 min [21–24]. Furthermore, the number of sessions and overall exposure to information through this method varies. It is important to note that lectures are not typically used as a solitary delivery method in TTT but in conjunction with others. Some have abandoned lecture-based teaching completely and have used the available training time for more engaging techniques [12, 25, 26].

Simulation-based learning has emerged as the preeminent method of teaching trauma care as it offers several advantages [27]. Multidisciplinary simulations have been shown to boost team performance and patient care [9]. It is a versatile method that allows for both technical and non-technical skill development in a safe and easily repeatable manner [28]. This technique allows for hands-on learning with high participant engagement [21]. Specific learning objectives can be reached by adjusting the complexity and context of the scenario [22]. However, it is only available to a limited number of participants [21]. Depending on the realism of the simulated scenario, three levels of fidelity have been described: High-, Medium, and Low-fidelity simulation, respectively [28]. Live Tisue Training (LTT) simulation using live animals has been used for surgical training purposes and offers high fidelity for technical skill development, but its role in TTT is not understood as there is no evidence to support its superiority to high-fidelity simulation using mannequins [29]. Simulation can be performed in an actual work environment, such as the trauma bay, called in-situ simulation, or in a simulation room, termed off-site simulation [22, 26]. In-situ simulation is most often reported in TTT as it offers several advantages, such as a high level of fidelity and a better understanding of the actual work environment [12, 23, 25, 26]. There are some concerns regarding safety, as these can disrupt workflow in the trauma bay when they are taking place and interfere with ongoing care [30]. As technology advances, its involvement in education is becoming more and more prominent. Virtual Reality (VR) simulation machines are emerging as the next step in training techniques for trauma care and may resolve some of the shortcomings of in-situ training while maintaining engagement and fidelity [31].

Providing feedback has been shown to increase knowledge retention significantly [12]. Feedback through debriefings is said to augment simulation learning outcomes by identifying and addressing participants’ knowledge gaps [32]. Several strategies have been employed, such as structured, video-assisted, or specific debriefing tools [19]. Debriefings are frequently used in the educational process related to TTT [12, 21–24, 26]. Studies reporting on TTT initiatives that incorporated video-assisted debriefings highlighted its positive impact on trainee learning experience over verbal feedback and its role in pinpointing specific correctable errors, suggesting its superiority as a teaching tool [23, 24, 26].

Trauma curriculums have been developed to cover the needs of highly functional, organized trauma systems. Typical training programs for medical professionals are ETC (European Trauma Course), ATLS (Advanced Trauma Life Support), and DSTC-DATC (Definitive Surgical and Anesthetic Trauma Care) [18]. ATLS has been the standard training curriculum for trauma care since 1978, endorsed by the American College of Surgeons Committee [20]. Perhaps the most popular, this program is taught in more than 60 countries and impacted the practice of more than a million physicians worldwide [33]. The learning experience is complex as it utilizes diverse interactive methods such as lectures, simulations of real-life scenarios, debriefings, and practical skills workshops, and is finalized with an assessment of skill acquisition after completion based on the employment of learned experience in a simulated scenario [20]. The European Resuscitation Council (ERC) developed a similar program, the European Trauma Course (ETC), in cooperation with several European medical societies such as the European Society for Emergency Medicine (EuSEM), the European Society for Trauma and Emergency Surgery (ESTES), and the European Society of Anaesthesiology (ESA), which has been in use since 2008 [34]. ETC aims to improve non-technical skills and is addressed to medical professionals and nurses, emphasizing the need to improve situational assessment, planning, task-sharing, decision-making, and communication [35]. It has been conceptualized as a multimodular training course with teaching done through workshops, skill stations, 30 progressive simulation scenarios, lectures, and demonstrations, with continuous assessment of participants [34]. Definitive Surgical Trauma Care (DSTC) is a training program endorsed by the International Association for Trauma Surgery and Intensive Care (IATSIC) in circulation since 1993, aimed at enhancing surgeons’ technical skills in wound management and damage control and aiding their decision-making process [36]. This program utilizes teaching methods such as lectures, surgical skill stations through LTT, and case discussion sessions. Definitive Anesthesia Trauma Care (DATC) has additionally been developed to enhance anesthesiologists’ skills and knowledge in damage control resuscitation and anesthesia [37]. This program can run separately or in conjunction with DSTC training. The joint DSTC-DATC course has additional benefits as it allows for the development of non-technical skills, such as efficient communication and information exchange between care providers [37]. The Definitive Preoperative Nurse Trauma Course (DPNTC) has been developed as a training module in DSTC training that addresses surgical nurses’ training needs in trauma surgical care in a multidisciplinary environment [36].

Despite the decades-long use of these programs, there is a scarcity of evaluation of their effects on patient outcomes [38]. These programs face scrutiny as there is debate about the cost-effectiveness balance [11]. Furthermore, there are difficulties in appropriating these curricula for low and middle-income countries (LMIC) [39]. Resource constraints are also an issue in rural areas where personnel availability is often limited [40]. Since the team consists of various staff members, there is concern about the relevance of the programs for nurses and auxiliary staff, and as such, new curriculums are currently under development [16].

Employment of ascertained curricula like ATLS has proved difficult in low and middle-income countries due to the cost of training medical professionals and the scarcity of trainers [38]. The applicability of such a program is reasonably limited in resource-constrained environments as it requires high tech such as computer tomography (CT) scans and bedside ultrasound machines to be readily available [41]. Additionally, team member roles and contributions in resuscitation vary significantly in LMIC as task shifting is common, and frequently, first responders to injury are not physicians [42]. For these reasons, many low-cost TTT programs have been developed. Amidst these, the Primary Trauma Care (PTC) course is the most popular and frequently reported in low-resource settings [38, 42]. It has been developed as a free course adapted from ATLS basic principles (emphasis on the primary and secondary surveys) and designed to be self-perpetuating and sustainable [41]. This training curriculum, first introduced in 1997, is endorsed by the WHO and is used in over 70 countries, with the manual available since 2003 in 14 languages [43]. Education is delivered using lectures, technical skill sessions, small group work sessions, and hands-on practice [41].

The Rural Trauma Team Development Course (RTTDC) has been compiled to improve trauma care in the low human resources environment and care capabilities of rural non-trauma hospitals by the American Colledge of Surgeons Committee on Trauma [44]. It aims to train small trauma teams (minimum three members) in coordinated assessment and management of severe, life-threatening injuries so that they can identify and transfer patients needing further treatment to a level I trauma center within the first 15 min of presentation [40]. This educational tool is composed of lectures and a communication module to increase the efficiency and accuracy of information exchange between care providers in a standardized manner [44].

There is heterogeneity between studies regarding educational intervention used, methods employed, and setting of the intervention. Most frequently reported are ATLS-based programs and proprietary trauma training algorithms derived from quality improvement program initiatives. Moreover, there is variability in the education delivery method used, as well as the frequency and duration of training programs, which can justify differences in study results. There is no study to report on ETC’s and DSTC-DATC’s impact on training performance measurements and patient outcomes, which warrants further investigation. Also, no study has evaluated PTC’s impact on in-hospital care and patient outcomes. We infer that an evaluation of available human and material resources should be performed before implementing any training program. Choosing the appropriate training program and education delivery method could be the key to achieving improved patient care and outcomes, and this should be based on the best evidence available. For this, we further elaborate on literature data about measurements of TTT performance and their improvement following different curricula in different settings.

## Trained team-related outcome measures

Trauma training aims to improve care by reducing the duration until critical intervention and decreasing mortality. As such, it focuses on helping participants better determine the nature and severity of lesions, prioritize care, promptly resuscitate and rapidly stabilize patients, and finally, coordinate timely transport to definitive care [45]. Assessing the impact of training on trauma teams is complex as there are many variables at play in the delivery of the above items, with many external factors involved in the direct outcomes of care. So far, there is no optimal measurement of TTT program’s performance and impact on patients [15]. Several outcomes have been considered (Table 2). Some studies reported trained team-related outcomes during resuscitation, measuring team-specific outcomes such as error rates (the appropriate time, the correct order, the correct frequency) in diagnostic procedures, resuscitation procedures, and communication, or self-reported outcomes such as team performance, collaboration, and knowledge [45]. The impact of training on staff plays a major role as perceived confidence and knowledge are key in an emergent situation, and involvement level is dependent on individual confidence in acquired skills [9]. As resuscitation is time-sensitive, measurement of team proficiency is done by measuring time-related outcomes, and several such measurements have been utilized, such as time to injury diagnosis, time to primary and secondary survey, time to intubation, times to CT scan, time to emergency department (ED) discharge, time to incision/operation room (OR), and total resuscitation time [21, 45]. All these represent the time elapsed from ER arrival to some specific intervention or test. Although it is important to perform certain interventions promptly, a fast time does not necessarily imply better patient care. Thus, these measurements should not be considered individually but should help paint a broader picture of training performance together with other evaluated criteria.

**Table 2:** Training outcomes measured in trauma

Criteria		Outcomes considered
Trained Team-related Outcomes	Team-specific outcomes	Error Rates
		Self-reported outcomes:
		Performance
		Collaboration
		Knowledge
	Time-related outcomes	Time to injury diagnosis,
		Time to primary and secondary surveys,
		Time to intubation,
		Time to CT scan,
		Time to ED discharge,
		Time to incision (OR) or other specific intervention.
		Total resuscitation time
Patient-specific outcomes measures		Mortality
		Morbidity
		Complication rate
		LOS

CT- Computer Tomography, ED- emergency department, OR- operation room, LOS- Length Of Stay

Evidence suggests that TTT positively impacts team-specific outcomes, but data is still limited. Steinemann and col. show how a complex crisis training course based on lectures, in-situ simulation, and video-assisted debriefing significantly improves team-specific outcomes such as teamwork (T-NOTECH scores from 16.7 to 17.7, *P* < 0.05) and task completion rates (from 48 to 62 participants, *p* < 0.001) [23]. Capella et al. show similar findings as TTT improved all aspects of team performance assessed, such as leadership, situation monitoring, mutual support, and communication (pre/post-test scores 2.87 vs. 3.46, *p* = 0.003, 3.30 vs. 3.91, *p* = 0.009, 3.40 vs. 3.96, *p* = 0.004, and 2.90 vs. 3.46, *p* = 0.001 respectively with overall improvement from 3.12 to 3.70, *p* < 0.001) [24]. Finally, ATLS training’s impact on these outcomes was showcased by Knoble et al., which illustrated how a high-frequency 24-month training program with monthly sessions increased participants’ self-confidence and knowledge [26]. Cohesion within a team, understanding one’s role, knowledge, and self-trust in performing certain tasks can have a domino effect on patient care and the emergency care system. For instance, TTT in the lower designated levels of care should empower care providers to effectively manage injured patients and adequately identify those in need of further treatment, thus helping reduce stress on oversaturated level I trauma centers. A recent study from a level I trauma center has shown that between 2019 and 2021, 19% of trauma transfers were of low severity and consequently discharged within 24 h [46]. This has significant effects on trauma teams as it utilizes unnecessary resources and distracts attention from where it is actually needed. RTTDC was designed for training in this setting, but its impact on team-specific outcomes has not been evaluated. A revision of training needs and appropriation of curricula is thus required for emergency staff that assess trauma patients in hospitals with other designations than level I.

**Fig. 1:**
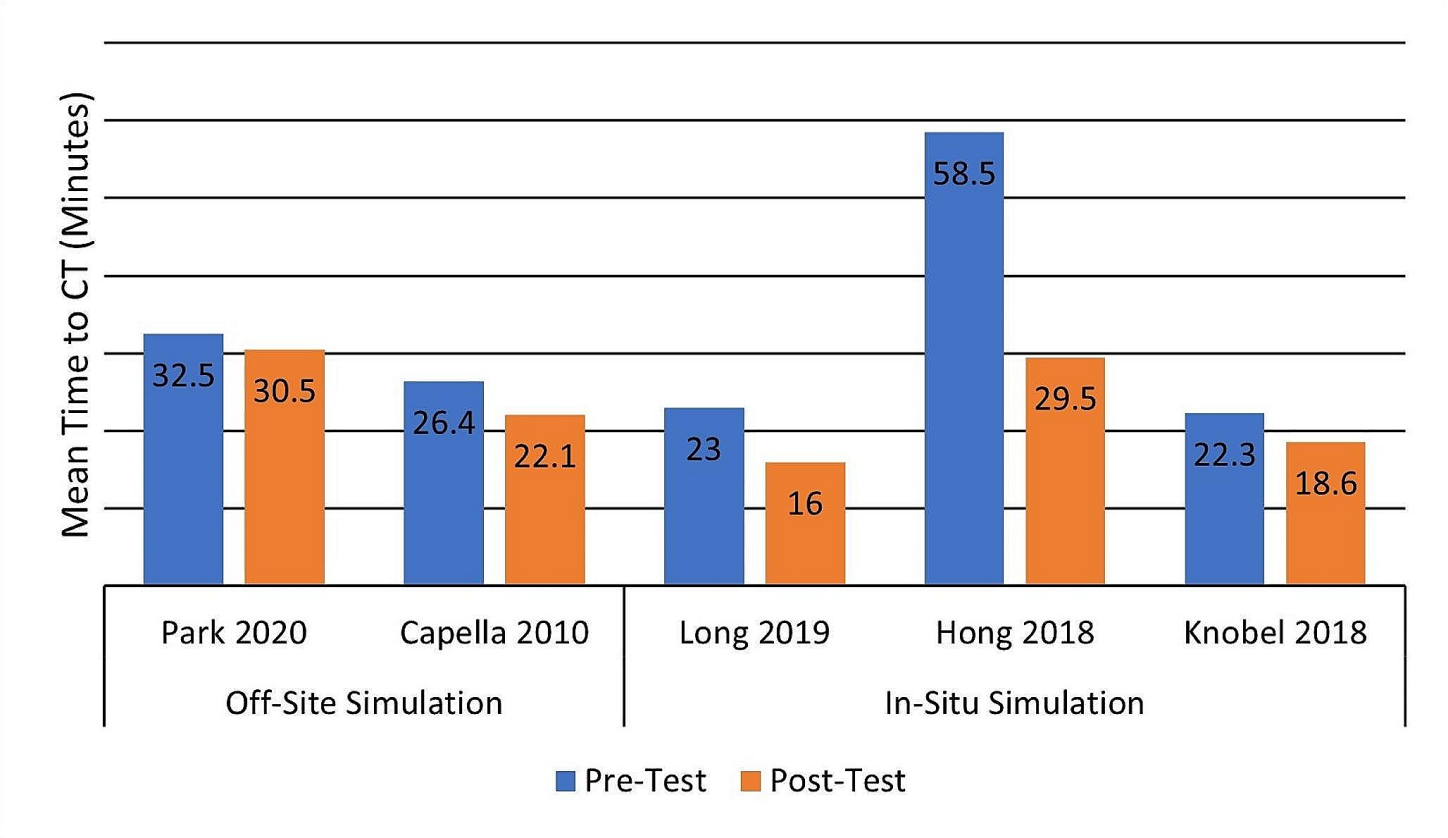
Simulation-based training impact on Time to CT (mean values are shown)

Time-related outcomes are most often used to evaluate trained team performance, and of these, time to CT evaluation stands out. Long et al. show that ATLS trauma training programs decreased time to secondary survey (14 to 6 min *P* = 0.004) and time to CT (23 to 16 min *P* = 0.01) in a cohort of 67 trauma patients from a level-1 trauma center [12]. A similar program using video-assisted debriefing and in situ simulations has shown a comparable effect with a significant reduction from 22.3 to 18.6 min (*P* = 0.001) from emergency department reception to CT [26]. However, Kristiansen and Col. performed a prospective interventional study on the effects of ATLS training on team performance and patient-related outcomes in a low-incidence level-2 regional hospital and found training did not improve trauma overall processing time and time to CT [22]. This may be due to the participation in the resuscitation of medical professionals who did not attend the training and perhaps due to the study design, as there was a long period after the education intervention to patient data collection. Another retrospective study showed that despite a lack of improvement in time to CT following ATLS training (32.5 to 30.5 min, *P* = 0.36), there was an improvement in important resuscitation procedure delivery like time to resuscitative thoracotomy, thoracostomy, and sheath placement [47]. In-situ simulation seems to yield better results related to team performance in trauma care, but to our knowledge, no study compares the two. For example, Fig. 1 depicts Pre/Post-Test differences in the mean time to CT reported for in-situ and off-site simulation training initiatives and shows a larger benefit gained through in-situ-based training. This observation needs to be thoroughly evaluated in further studies.

Other training programs also show improvements in team performance in terms of time-related outcomes. Hong et al. evaluated time to specific tests or procedures in critical care of trauma patients before and after TTT and found improved time to blood tests, time to initiation of hemostatic procedures, time to CT, and administration of Tranexamic Acid following training (13 vs. 8 min, *P* < 0.01, 113.5 vs. 31.0 min, *P* = 0.01, 58.5 vs. 29.5 min, *P* = 0.01, and 90 vs. 31 min, *P* < 0.01 respectively) [25]. Capella et al. show a significant improvement in time to CT, intubation, and time to operation room (OR) (26.4 vs. 22.1 min, *P* = 0.005, 10.1 vs. 6.6 min, *P* = 0.049, and 130.1 to 63.8 min, *P* = 0.021 respectively) after seven sessions of a TeamSTEPPS intervention followed by simulations [24]. However, it is unclear whether this improvement in team performance correlates to improved patient outcomes as they show no significant reduction in patient mortality, complication, and length of stay. Steinmann’s study also shows that despite improvements in teamwork and task completion scores, training had no effect on patient mortality, morbidity, and LOS [23]. Similarly, a large retrospective study on a TTT four-year program showed that despite improvements in trained team outcomes, there was no improvement in patient outcomes such as mortality, but rather an increase in ED LOS (Pre-Test 4.88 h vs. Post-Test 7.17 h, *P* < 0.001) [21].

Two studies have assessed RTTDC’s impact on trained team performance. First is a longitudinal multicenter cohort study by Kappel et al. that assessed time-related outcomes in the care of injured patients from level III and IV trauma centers [44]. They compare the performance of participants from 16 hospitals that either did not receive any trauma team training or received training with or without an additional communication module. Time-related outcomes significantly improved in the trained cohort, and further, the participants who received additional communication training had significantly reduced times to transfer squad arrival and patient hand-out compared to the cohort receiving only RTTDC training (114.35 vs. 95.72 min exposed only to RTTDC and 77.17 min exposed to RTTDC and communication module, *P* < 0.05, respectively for time to squad arrival, and 31.12 min for RTTDC and communication module vs. 67.19 min for RTTDC cohort, *P* < 0.01 respectively for time to patient hand-out). A further study by Malekpour et al. has shown similar results regarding time to transfer acceptance and total transfer times (139.2 vs. 110 min, *P* = 0.003 and 257.3 vs. 219.2 min, *P* = 0.002, respectively) [40]. Yet, these improvements did not significantly impact patient outcomes such as LOS, complication rate, or mortality.

## Patient-related outcome measures

Improvement of patient outcomes is, however, the primary goal of training, and a direct change in mortality, morbidity, complication rate, and length of stay (LOS) has been proposed as a measurement of training results, although these outcomes are rarely reported [15]. Other outcomes have thus been considered as surrogates for directly observed patient outcomes, such as the time to critical intervention (time to intubation, time to CT, time to OR), which are known to correlate with mortality and morbidity [21, 27].

**Fig. 2:**
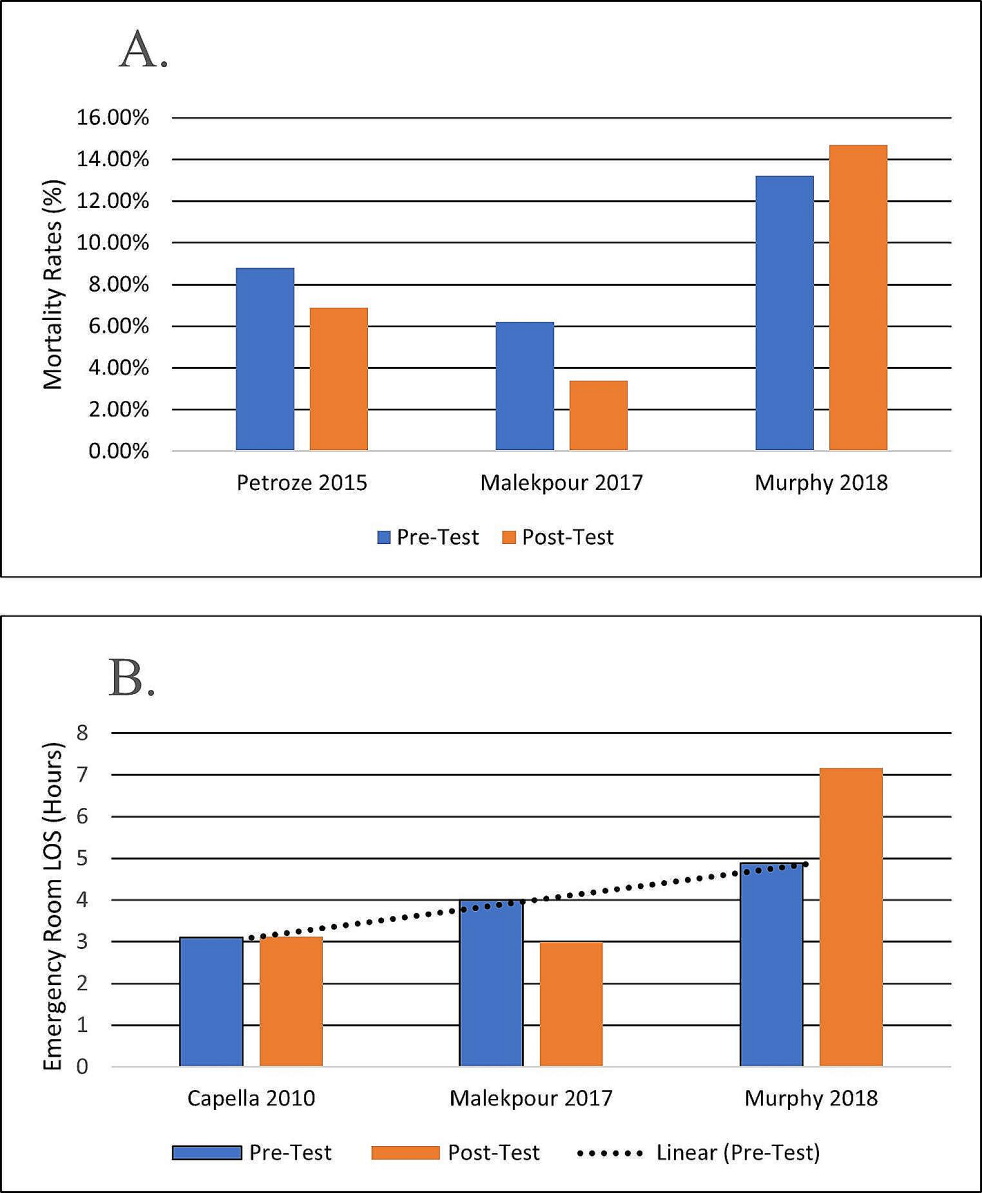
Pre/Post trauma training patient-related outcomes measures. **(A)** Training impact on mortality rates; **(B)** Training impact on emergency room Length of Stay (LOS) with trendline for pre-interventional measures (mean values are shown)

There is debate about the utility of trauma training programs and their benefits on patient outcomes. A meta-analysis comparing the cost-benefit of training and patient outcomes has shown that TTT’s impact on patient outcomes is poor [11]. Another recent meta-analysis reporting on nine studies about TTT and patient outcomes has shown a lack of improvement in trauma mortality [15]. Studies reported consistently a lack of statistically significant improvement in patient mortality rates [13, 23, 24, 40, 48]. Mortality in trauma is multifactorial and may not be related to care. Yet some identified a mortality rate reduction, though of no statistical significance (Fig. 2A) [24, 48]. Increasing the patient cohort or an increase in injury severity might have yielded a statistically significant decrease in mortality [24]. One study shows a significant mortality rate reduction in the subgroup of patients with severe injuries from 58.51 to 37.10%, *P* = 0.009 [48]. These gains appear marginal, yet a drop of 20% in overall trauma mortality has been observed globally from 2000 until 2019, particularly in countries where TTT is often performed, such as the European Countries [1]. This justifies efforts to conduct and improve trauma team training as its effects may take longer to consolidate and show measurable outcome improvements.

Similarly, there is a consistent lack of significant reduction of patient LOS being reported. One study also suggests that there was an increase in patient LOS following TTT educational intervention, but this may be due to overcrowding and understaffing of the ED during the data collection timeframe in the post-intervention period [21]. The graph in Fig. 2B plots LOS in emergency departments as reported in the literature related to TTT, and an increasing trend can be observed. Time in the ED is multifactorial and is related to human resources availability and other factors unrelated to trauma care. This implies that improving such outcomes is often beyond TTT’s reach as external factors such as overcrowding cannot be resolved necessarily by these means. Overcrowding in emergency rooms is becoming more and more a prominent issue [49]. Also, improving some of the infrastructure and workflow issues may have a ripple effect on patient LOS that is larger than what trauma training can achieve. Some studies identified areas of vulnerability as an effect of training programs, such as the misplacement of medical devices and lack of knowledge regarding staff positioning, which disrupted workflow and prompted further protocol improvement [12, 22]. These improvement efforts in trauma protocols are acclaimed, and the need for a tool to assess in-situ team performance is crucial for identifying those areas of vulnerability in care delivery.

## Reported study limitations

The level of evidence is currently low as most studies are retrospective cohort studies with very few prospective interventional studies reported. So far, no randomized clinical trial has evaluated TTT and patient outcomes, but one multicentric randomised trial is currently ongoing [38].

Despite the evidence to support that TTT translates to team performance improvements, there is still a lack of association with significant patient outcomes. Reasons for the lack of correlation with significant outcomes exist. Measuring outcomes is perhaps the major difficulty of this field of research, as patient outcomes in trauma depend on multidisciplinary care and are multifactorial [29]. Most findings are limited by the study design and execution [32]. Securing consent from trauma patients is a significant impediment in data collection for outcome assessment in a prospective design [45]. A small sample size was reported in several studies [12, 22, 24]. The Hawthorne effect on study results was assessed in some instances [12, 26]. Regular rotation of staff during shifts makes predictions of impact on patient outcomes difficult as there is no way of knowing how many participants in resuscitation (if any) were involved in the training session [22, 24, 26, 44]. The team is put together on short notice and has a variable composition that is different from the simulated scenarios, which further influences the results [21]. Skill decay may contribute to the lack of observed effect, and the needed refreshment TTT frequency is unknown [22]. It is unclear whether previous exposure to trauma resuscitation might be the reason for the improvement in team performance or whether the post-interventional exposure to practice improved participants’ skills rather than the intervention itself [23, 24]. Simulation improved trained team-related outcomes. However, often, due to time constraints and the complexity of the program, it was difficult to involve all personnel in the immersive simulation experience, which implies the positive effect observed could be due to the training of key individuals [12]. Finally, overcrowding may affect workflow in the ED and thus influence patient care outcomes such as LOS [21]. 

## Conclusions

The trauma system is complex, dependent on the available infrastructure, and with care provided at several stages by heterogeneous teams. The outcome in trauma is thus multifactorial, and literature data highlights the importance of optimizing training for each step of care and individualizing it to the specific needs of participants. Trauma team implementation has been a breakthrough in the management of trauma. Many curriculums have been developed, and several methods have been employed to train the myriad of medical professionals involved in efficient trauma care. There is evidence that TTT improves team performance and that skills developed in training translate into medical practice. However, a definitive impact on meaningful patient outcomes eludes. ATLS training courses have propelled trauma care further, but development has since stagnated, with additional gains seeming marginal. Global mortality rates in trauma have a downward trend; thus, the effect of training might not be immediately visible, and larger cohort studies are needed to evaluate TTT impact over time. Other curricula have been proposed, but their performance has not been assessed so far, and there is a current race to provide evidence of their efficiency in improving patient outcomes. Furthermore, novel educational delivery methods have emerged, and the aid of technology, such as virtual reality machines, may resolve some of the limitations of simulation-based learning. Our study emphasizes the importance of trauma team implementation and training and provides literature data that guides toward optimal education delivery.

## Data Availability

No datasets were generated or analysed during the current study.
